# CHMP4C as a novel marker regulates prostate cancer progression through cycle pathways and contributes to immunotherapy

**DOI:** 10.3389/fonc.2023.1170397

**Published:** 2023-06-14

**Authors:** Hongtuan Zhang, Dongze Liu, Zheng Qin, Bocun Yi, Liang Zhu, Shengxian Xu, Kaibin Wang, Shaobo Yang, Ranlu Liu, Kuo Yang, Yong Xu

**Affiliations:** Institute of Urology, the Second Hospital of Tianjin Medical University, Tianjin, China

**Keywords:** CHMP4C, prostate cancer, diagnostic and prognostic biomarkers, accurate treatment, anti-tumor

## Abstract

**Background:**

CHMP4C is one of the charged multivesicular protein (CHMP), and is involved in the composition of the endosomal sorting complex required for transport III (ESCRT-III), facilitating the necessary separation of daughter cells. CHMP4C has been proposed to be involved in the progression of different carcinomas. However, the value of CHMP4C in prostate cancer has not yet been explored. Prostate cancer is the most frequently occurring malignancy among male and remains a leading cause of deaths in cancers. So far, clinical therapy of prostate cancer is more inclined to molecular classification and specific clinical treatment and research. Our study investigated the expression and clinical prognosis of CHMP4C and explored its potential regulatory mechanism in prostate cancer. The immune status of CHMP4C in prostate cancer and relative immunotherapy were then analyzed in our study. Based on CHMP4C expression, a new subtype of prostate cancer was established for precision treatment.

**Methods:**

We studied the expression of CHMP4C and relative clinical outcome using the online databases TIMER, GEPIA2, UALCAN, and multiple R packages. Meanwhile, the biological function, immune microenvironment and immunotherapy value of CHMP4C in prostate cancer were further explored on the R software platform with different R packages. Then we performed qRT-PCR, Western Blotting, transwell, CCK8, wound healing assay, colony formation assay and immunohistochemistry to verify the expression of CHMP4C, carcinogenesis and potential regulatory mechanisms in prostate cancer.

**Results:**

We found that the expression of CHMP4C is significant in prostate cancer and the high expression of CHMP4C represents a poor clinical prognosis and malignant progression of prostate cancer. In subsequent vitro validation, CHMP4C promoted the malignant biological behavior of prostate cancer cell lines by adjusting the cell cycle. Based on CHMP4C expression, we established two new subtypes of prostate cancer and found that low CHMP4C expression has a better immune response while high CHMP4C expression was more sensitive to paclitaxel and 5-fluorouracil. Above findings revealed a new diagnostic marker for prostate cancer and facilitated the subsequent precise treatment of prostate cancer.

## 1 Introduction

In male population, prostate cancer is still the 2nd most commonly diagnosed tumor worldwide. ​There were approximately 1,400,000 new cases and 375,000 deaths in 2020 and the incidence has been increasing worldwide (1) (2). Prostate cancer is geographically most prevalent in the Nordic population and is associated with many risk factors such as family history, race and hereditary syndromes (3). The high degree of heterogeneity in prostate cancer treatment decisions and outcomes dictates appropriate risk stratification of patients. This requires that we distinguish between the relatively benign state of prostate cancer and the more aggressive state, so the inclusion of prognostic and predictive biomarkers of clinical value is urgent. The progression and development of prostate cancer are complex and heterogeneous, with approximately 20-30% of male patients with limited prostate cancer recur after treatment and a 5-year survival rate of only 30% when metastases occur. More importantly, the therapy of male patients with metastatic and castration-resistant prostate cancer remains unsatisfactory. Immunotherapy is currently a hot topic in prostate cancer treatment. In immunotherapy for prostate cancer, cytotoxic T lymphocyte-associated antigen 4 (CTLA4), programmed death ligand-1 (PD-L1), and programmed death-1 (PD-1) inhibitors have shown promising outcomes in terms of anti-tumor immune therapy. However, some clinical tests on immunotherapy in prostate cancer patients have not been as effective as expected. In the era of precision medicine, specific and targeted treatment for different tumor subtypes of patients is considered to be the best measure to achieve the maximum therapeutic effect (4) (5). Therefore, exploring and establishing new subtypes of prostate cancer will help to address above issues and improve the clinical prognosis of patients.

Chromatin modifying protein 4C (CHMP4C), one of the chromatin modifying protein (CHMP), is a constituent of the endosomal sorting complex needed for transport (6) (7). CHMP4C transports the required endosomal sorting complex involved in cell division of daughter cells (8) and plays a greatly significant role in many processes such as cancer pathogenesis and progression in the form of extracellular vesicles (9). CHMP4C has been shown to have a regulatory effect in numerous tumors, including human ovarian cancer (10), lung cancer (11) (12) and cervical cancer (13). However, the role of CHMP4C in prostate cancer is rarely mentioned. Previous studies have shown that CHMP4C is abundantly expressed as a component of extracellular vesicles in patients with high Gleason scores and as a novel signature of pyroptosis that affects the prognosis of prostate cancer patients (14) (15). Therefore, further validation of CHMP4C expression in prostate cancer, and whether CHMP4C affects the biological behavior of prostate cancer and the regulatory pathways involved, will help us to identify new biomarkers and new therapeutic options.

## 2 Materials and methods

### 2.1 Online database and R packages for analyzing CHMP4C expression

The timer database (TIMER2.0 (cistrome.org)) was applied to compare the differences in the expression of CHMP4C between prostate tumor samples and normal tissue samples (16). Prostate cancer transcriptome data were got from the TCGA database (https://cancergenome.nih.gov/) and used for validation of the difference analysis and paired difference analysis with “ggpubr” and “limma” packages (17).

### 2.2 Online database and R packages for analysis of CHMP4C clinicopathological correlations

The GEPIA2 database (http://gepia2.cancer-pku.cn/#analysis), “survival” package and “survminer” package were applied to analyze the prognosis of CHMP4C in prostate cancer (18). UALCAN database (UALCAN (uab.edu)) was applied to find the relationship between CHMP4C expression and prostate cancer Gleason score, lymph node metastasis status and TP53 mutation status (19) (20).

### 2.3 Biological functional analysis of CHMP4C

To further investigate the regulatory role of CHMP4C in cell proliferation, we performed co-expression gene analysis and GSEA analysis using the “limma” package and “enrichplot” package and identified important regulatory roles of CHMP4C in the cell cycle. To deepen our understanding of the biological functions of CHMP4C, we conducted grouping differences analysis of CHMP4C using the “limma” package and performed KEGG and GO analysis based on the grouping results. KEGG and GO gene sets were got from the Gene Set Enrichment Analysis (GSEA) website (GSEA (gsea-msigdb.org) (21). Then the R packages ‘enrichplot’, ‘ggplot2’, ‘clusterProfiler’ and “org.Hs.eg.db” were utilized to perform the GO and KEGG analysis.

### 2.4 Immune infiltration analysis of CHMP4C in prostate cancer

limma package, estimate package and reshape2 package were used to analyze the immune and mesenchymal scores of CHMP4C in prostate cancer. CIBERSORT is a deconvolution algorithm that can be used to transform the normalized gene expression array into the composition of infiltrating immune cells. Based on the result of CIBERSORT, we obtained the relationship of CHMP4C with the infiltration level of 22 immune cells using “limma” packages. We then explored the relationship of CHMP4C expression with 49 immune checkpoint genes with “ggplot2” and “reshape2” R packages and set the p-value filter to 0.001.

### 2.5 Analysis of the potential therapeutic value of CHMP4C in prostate cancer

IPS-CTLA4 blocker and IPS-PD1/PD-L1 blocker data for prostate cancer from TCGA were got in TCIA (https://tcia.at) and utilized to predict the response to ICI in patients with low and high expression of CHMP4C. External independent immune validation queue data from GEO database (https://www.ncbi.nlm.nih.gov/geo/, GSE67501). The “pRRophetic” package was applied to predict the drug sensitivity of CHMP4C.

### 2.6 Cell culture

The prostate cancer cell lines PC-3 and DU-145 were got from the Affiliated Cell Resource Center of the Chinese Academy of Medical Sciences. Culture medium was RPMI1640 medium (Biological Industries) with 10% added fetal bovine serum (Biological Industries) and 1% penicillin and streptomycin (100units/ml, Solarbio). The environmental conditions for incubation were 37 degrees, 5% CO2 humidified incubator.

### 2.7 Quantitative real-time PCR and transfection of si-RNA molecules

Total RNA Kit (Omega Bio-Tek, USA) was used to isolate and purify total RNA from cell lines, and BIOG cDNA Synthesis Kit (BioDai, Changzhou, China) was used for reverse transcription.

In qRT-PCR, GAPDH primers were designed as follows (GAPDH-F: GGAAGGTGAAGGTCG GAGTCA, GAPDH-R: GTCATTGATGGCAACAATATATCCACT) and CHMP4C primers were designed as follows (CHMP4C-F: AGACTGAGGAGATGCTGGGCAA, CHMP4C-R: TAGTGC CTGTAATGCAGCTCGC). Relative expression differences were calculated using the 2-ΔΔCt method with the GAPDH gene as a control. si-NC and si-RNA of CHMP4C were designed as follows (si-NC: CCUCUGGCAUUAGAAUUAUTT, si- CHMP4C: CCUGCGUCUC UACAACUAU).

### 2.8 Western blot

RIPA buffer with PMSF was used to get the total protein, and the total protein concentration was determined with the BCA method (solarbio, Beijing, China). The protein was separated on 10% SDS/PAGE gels and transferred to PVDF membranes. After transferring, the membranes were blocked with 5% skim milk and incubated overnight in primary antibody at 4°C. Bound antibodies were detected by horseradish peroxidase-labeled secondary antibody. Western blot analysis was performed with ECL luminescent reagent (solarbio, Beijing, China). The antibodies were displayed as follows (CHMP4C: Abcam, Cambridge, UK, ab168205, CDK2: Abcam, Cambridge, UK, ab32147, CCNA2: Abcam, Cambridge, UK, ab181591, GAPDH: Abcam, Cambridge, UK, ab8245).

### 2.9 Immunohistochemistry

Pathology slides from patients with prostate cancer and benign prostate hyperplasia (BPH), and we cut paraffin sections of the corresponding tissues. Immunohistochemical antibodies were obtained from Abcam, Cambridge, UK, ab272638. DAB reagent was used for staining (Zhongshan Jinqiao, ZLI-9018).

### 2.10 Cellular functional assays

In CCK8 assay, si-NC and si-CHMP4C cells were inoculated into 96-well plates at a density of 2 × 103 cells/well, incubated at 37 degrees for 3 hours, and absorbance was measured at 450nm for 3 consecutive days. In the colony formation assay, 2 groups of cells were inoculated in 6-well plates at a density of 1000 cells/well and cultured until visible colonies were formed. Cell colonies were fixed in 4% paraformaldehyde (Solarbio, Beijing, China) for 20 min and then stained with 0.1% crystal violet solution (Solarbio, Beijing, China) for 20 min. In the wound healing assay, we inoculated cells on 6-well plates and cultured cells until fusion reached 80%-90%, using pipette tips for cell scoring and PBS for cell rinsing. Photographs were taken under the microscope at 15h, 30h, and 45h, respectively. In the transwell assay, the matrigel was melted and spread in a 24-well transwell chamber. The lower chamber was added with 10% fetal bovine serum. After 12 h of starvation, cells were transferred to transwell chambers and incubated at 37° C for 36 h. The remaining cells in the upper chamber were erased with cotton swabs and fixed with paraformaldehyde for 20 min. The cells were stained with 0.1% crystal violet solution for 20min.

### 2.11 statistical analysis

Statistical analysis of bioinformatics was conducted on the R software platform and experimental data were counted using GraphPad Prism version 9.0. We adopted a t-test to compare differences between the two groups and all statistical tests were set as two-sided (P<0.05 was considered statistically significant. ***, **, * stood for P value <0.001, P value <0.01, P value <0.05, respectively).

### 2.12 Ethics declaration

All studies involving human tissues in this study have been reviewed and approved by the Medical Ethics Committee of the Second Hospital of Tianjin Medical University (KY2023K038), and all experiments were conducted in accordance with relevant requirements and guidelines.

## 3 Results

### 3.1 High expression of CHMP4C in prostate cancer

**Figure 1:**
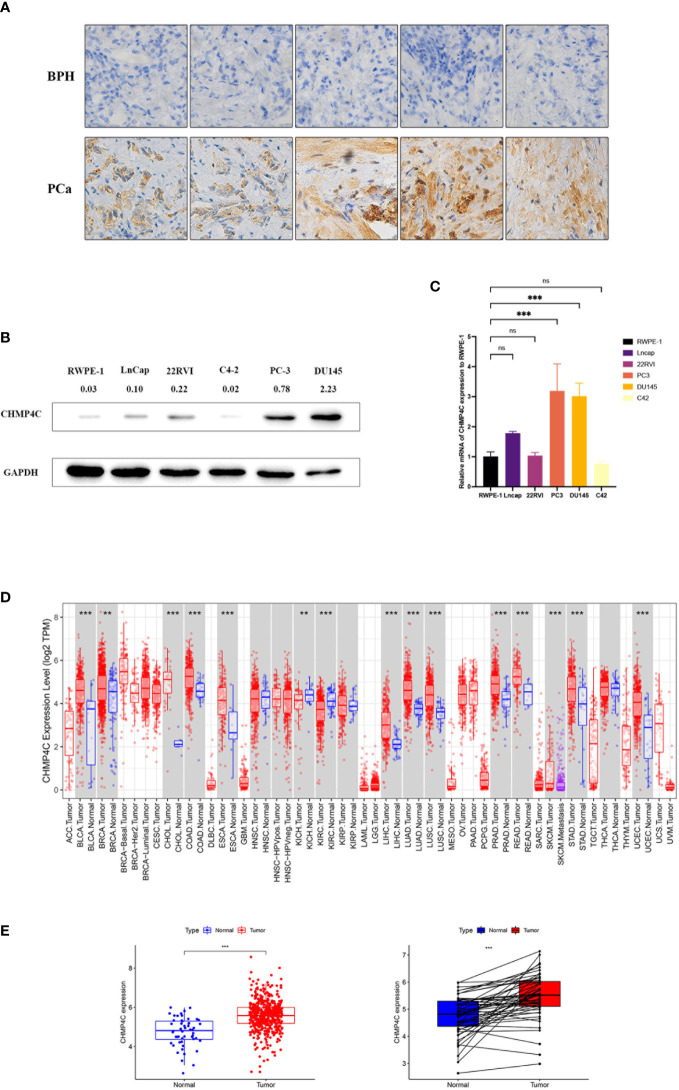
CHMP4C expression in prostate cancer. Immunohistochemical staining of CHMP4C in prostate cancer and benign prostatic hyperplasia **(A)**. Western blot of CHMP4C in cell lines **(B)**. qRT-PCR of CHMP4C in cell lines **(C)**. Validation of CHMP4C expression in timer online **(D)**. Validation of CHMP4C expression in the TCGA database **(E)**. ***, **, ns stood for P value <0.001, P value <0.01, P value >0.05, respectively.

We first performed immunohistochemical staining on prostate cancer tissues and benign prostatic hyperplasia tissues, revealing that CHMP4C was higher in prostate cancer tissues (**Figure 1A**). To select appropriate cell lines for a subsequent cell functional experiment, we tested the expression of CHMP4C in six prostate cancer cell lines (RWPE-1, LnCap, 22RVI, C4-2, PC-3, DU145). The results showed that the expression levels of PC-3 and DU-145 were the highest compared to RWPE-1. (**Figures 1B, C**). Then the level of CHMP4C expression was verified again by the timer database. CHMP4C was shown to be hyper-expressed among most cancer types, especially prostate cancer (**Figure 1D**). Next, we performed variance analysis and paired difference analysis using prostate cancer data from the TCGA database, with results consistent with those described above (**Figure 1E**). All of the results reveal that CHMP4C is a potential diagnostic biomarker for prostate cancer.

### 3.2 Correlation of CHMP4C expression with prostate cancer clinicopathology and prognosis

**Figure 2:**
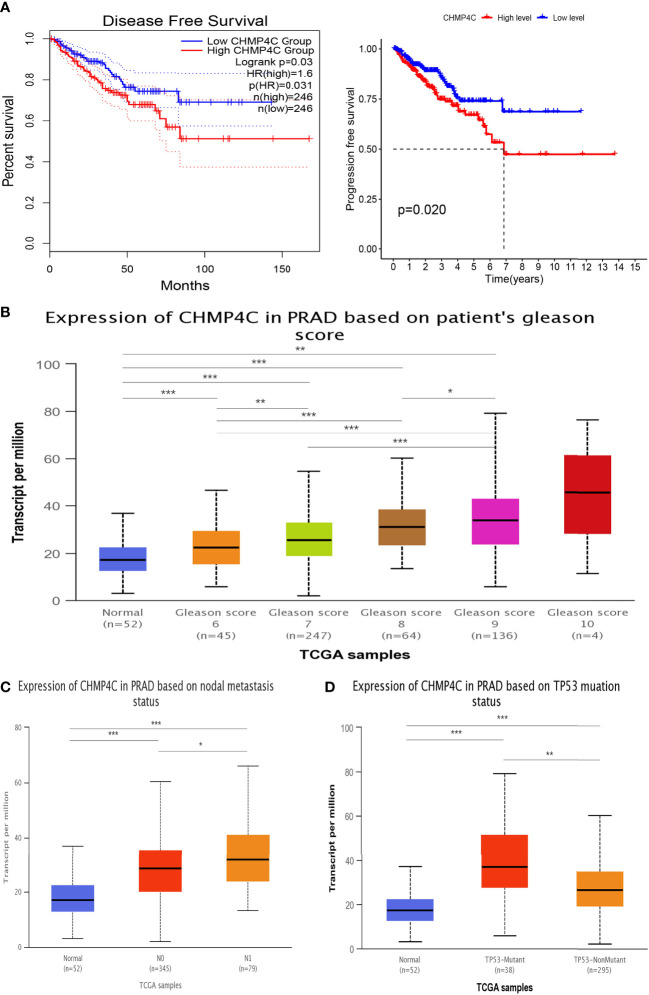
Association between CHMP4C and clinicopathological features. Survival analysis of CHMP4C **(A)**. Correlation of CHMP4C with the gleason score **(B)**, lymph node metastasis **(C)**, and TP53 mutation status **(D)**. ***, **, * stood for P value <0.001, P value <0.01, P value <0.05, respectively.

In this study, we utilized the GEPIA2 database and the packages “survival” and “survminer” to explore the correlation of CHMP4C expression levels with prognosis to determine whether CHMP4C can be regarded as a diagnostic biomarker for prostate cancer. We found that up-regulated CHMP4C was generally accompanied by a poor prognosis in terms of DFS and PFS (**Figure 2A**). Next, CHMP4C expression in prostate cancer was investigated in different pathological parameters using the UALCAN database. Regarding Gleason score, the expression of Gleason6/7/8/9 was significantly upregulated compared to normal controls. Although no significant difference was found in CHMP4C expression in Gleason10 samples, this may be due to the small sample size in this category (n=4). Future studies with larger sample sizes may clarify the role of CHMP4C in high-grade prostate cancer (**Figure 2B**). In lymph node metastasis, CHMP4C expression gradually increased as the number of lymph node metastases increased (**Figure 2C**). Similarly, CHMP4C expression was also upregulated in both TP53 mutant and non-mutant compared to normal control (**Figure 2D**). Taken together, the findings suggested that CHMP4C was indeed correlated with prostate cancer progression and invasion.

### 3.3 CHMP4C *in vitro* validation of DU-145 and PC-3 cell lines

**Figure 3:**
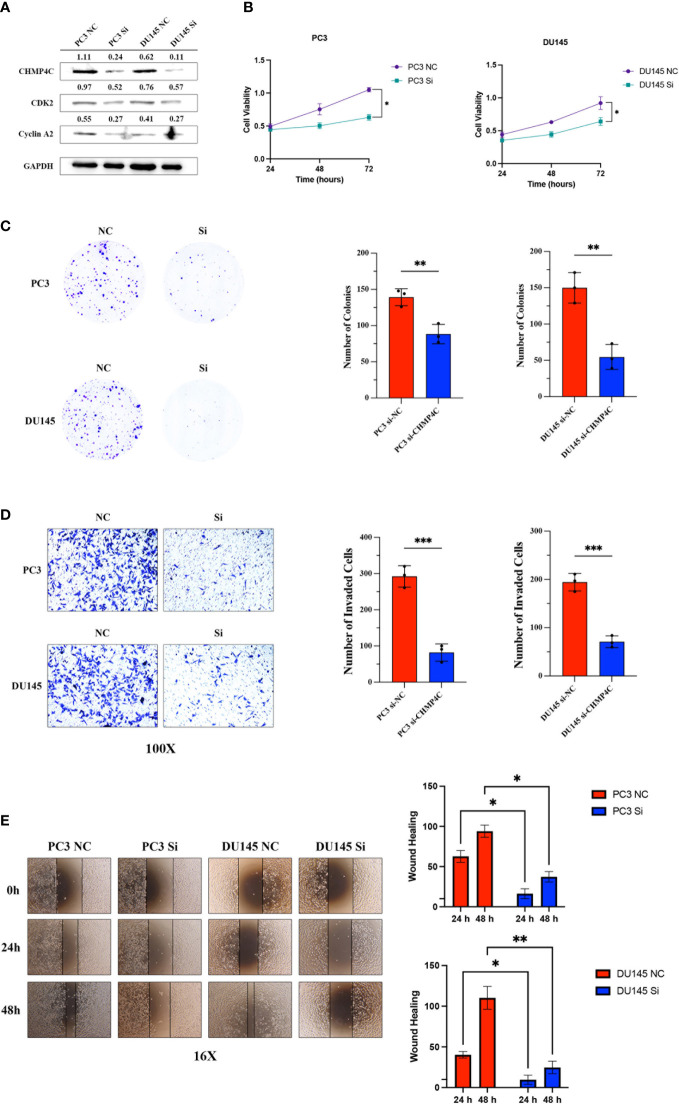
CHMP4C promoted the proliferation, invasion, and metastasis of PC-3 and DU-145 cell lines *in vitro*. Knockdown efficiency of CHMP4C and changes of downstream proteins CDK2 and cyclinA2 **(A)**. CHMP4C knockdown inhibited the migration of CHMP4C inhibited PC-3 and DU-145 cell proliferation tested by CCK-8 assay **(B)**. Knockdown of CHMP4C inhibited the colony formation ability of PC-3 and DU-145 cells **(C)**. Transwell invasion assays were conducted in PC-3 and DU-145 cells **(D)**. Knockdown of PC-3 and DU-145 cells migration **(E)**. ***, **, * stood for P value <0.001, P value <0.01, P value <0.05, respectively.

PC-3 and DU-145 cells were transfected with 50 nM si-CHMP4C or si-con using Lipofectamine 2000. After 48 h, the cells were harvested for analysis. The expression level of cells was detected by Western blot. The results suggested that the CHMP4C protein expression levels of PC-3 and DU-145 were significantly decreased after si-CHMP4C transfection (n=3, t-test, p<0.05) (**Figure 3A**). We then performed a cellular function experiment to investigate whether CHMP4C induces prostate cancer cell malignancy. CCK-8 cell proliferation assay results showed that CHMP4C knockdown reduced PC-3 and DU-145 proliferation viability compared to controls (n=3, t-test, p<0.05) (**Figure 3B**). Similar conclusions have also been verified in colony formation experiments. We found a significant decrease in the number of si-CHMP4C clones compared to the si-con group (n=3, t-test, p<0.01) (**Figure 3C**). In the invasiveness assay transwell, the number of invading and metastatic cells of PC-3 and DU-145 transfected with si-CHMP4C was reduced compared to the control (n=3, t-test, p<0.001) (**Figure 3D**). In the wound healing assay, knockdown of CHMP4C would further diminish the migration distance of PC-3 and DU-145 (**Figure 3E**). The above results suggested that CHMP4C is a positive factor of prostate cancer cell proliferation and invasion.

### 3.4 Co-expression analysis of CHMP4C in prostate cancer

**Figure 4:**
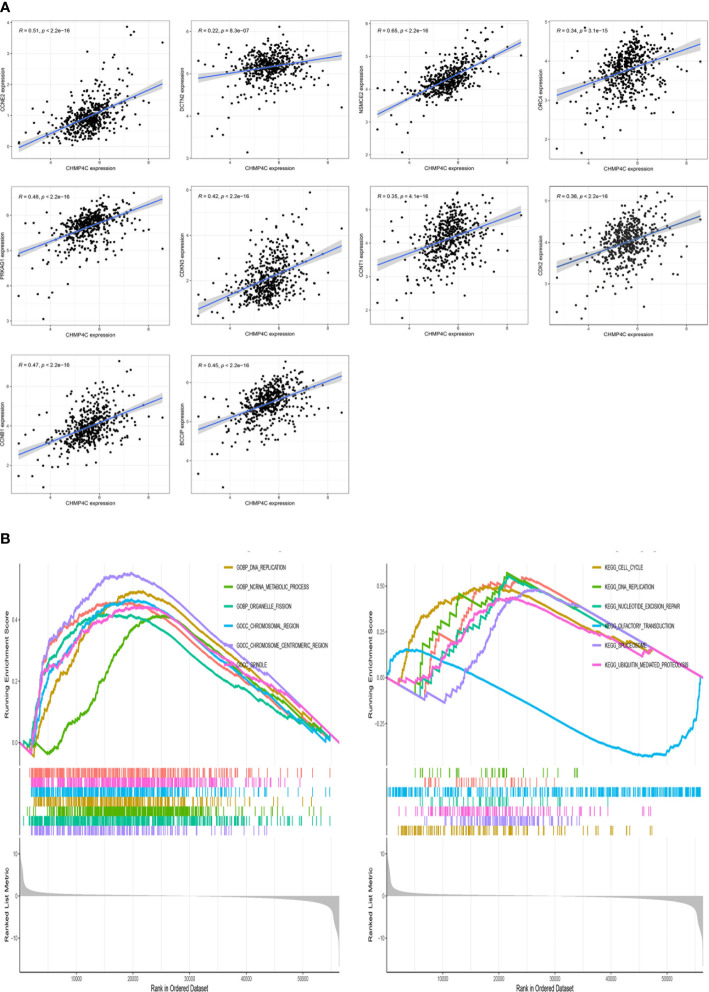
CHMP4C is involved in the cell cycle regulation of PC-3 and DU-145. CHMP4C co-expressed genes associated with the cell cycle **(A)**. GSEA analysis of CHMP4C in prostate cancer **(B)**.

In order to further investigate the possible mechanism of action of CHMP4C in prostate cancer, we performed a co-expression analysis of CHMP4C using the TCGA database (**Supplementary Table 1**). The results of the co-analysis suggested that CHMP4C was positively correlated with cell cycle-related genes including CCNE2, DCTN2, NSMCE2, ORC4, PRKAG1, BCCIP, CDKN3, CCNT1, CDK2, CCNB1 (**Figure 4A**). GSEA results also showed that CHMP4C was mainly involved in DNA replication in GOBP and in cell cycle regulation in KEGG (**Figure 4B**). Cyclin-dependent kinases (CDKs) and associated cell cycle chaperone proteins are the main regulators of the cell cycle. The CDK2 and cyclinA2 proteins have been shown to play a major role in the regulation of the cell cycle by regulating the transition from the G1 to the S phase. Our experimental results showed that CHMP4C knockdown resulted in a significant decrease in the expression of CDK2 and CyclinA2 (**Figure 3A**). The above findings showed that CHMP4C is likely to be involved in regulating the prostate cancer cell cycle.

### 3.5 Analysis of related biological functions of CHMP4C

**Figure 5:**
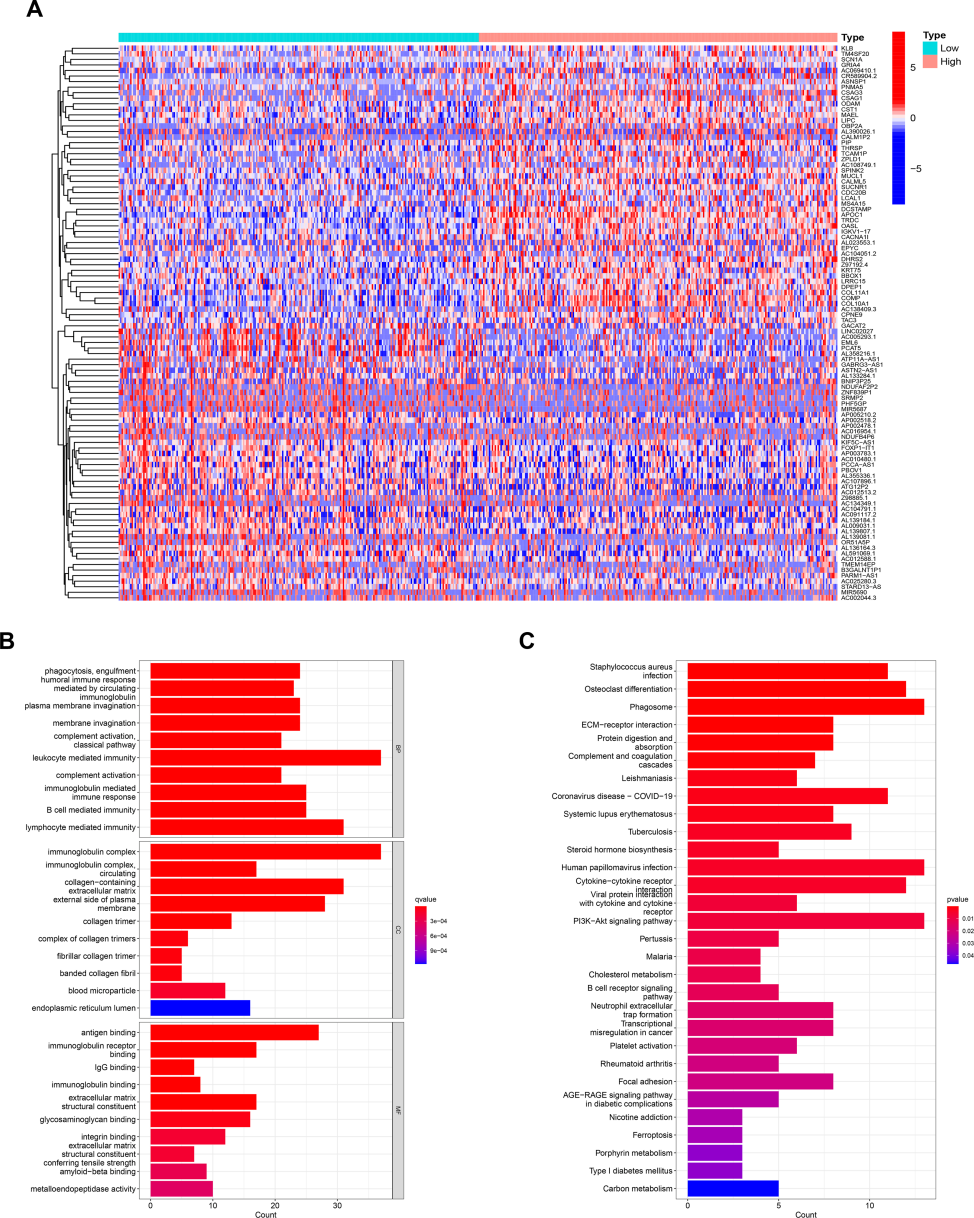
Analysis of related biological functions of CHMP4C. Differential genes grouped by CHMP4C **(A)**. GO analysis of CHMP4C **(B)**. KEGG analysis of CHMP4C **(C)**.

We performed a grouped variance analysis of CHMP4C and presented it with a heat map (**Figure 5A**). Then GO and KEGG function enrichment analysis were conducted for these differential genes. The results suggested that the GO analysis was mostly related to the regulation of immune function (**Figure 5B**). KEGG analysis also suggested that these genes are also involved in regulating immune function. (**Figure 5C**). The above findings suggested the potential value of our CHMP4C in the mediation of immune function.

### 3.6 Differential analysis of CHMP4C immune cell infiltration

**Figure 6:**
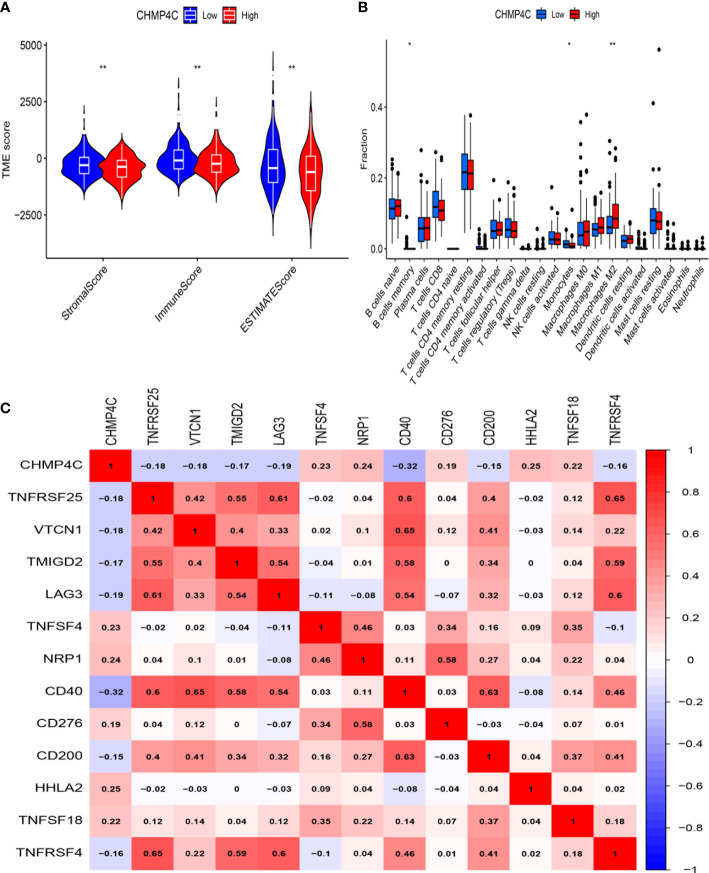
Immune microenvironment analysis of CHMP4C. Tumor microenvironment (TME) scores of CHMP4C **(A)**. 22 immune cell infiltration analysis of CHMP4C **(B)**. Correlation analysis between CHMP4C and immune checkpoints **(C)**. **, * stood for P value <0.01, P value <0.05, respectively.

To further investigate how CHMP4C relates to immune function, we explored the correlation between the ratio of immune and mesenchymal components and the expression of CHMP4C. Next, the estimate score, stromal score, immune score of the low and high expression groups of CHMP4C were evaluated. The results suggested that low expression of CHMP4C had a higher score (**Figure 6A**, p<0.01). The correlation between CHMP4C and 22 infiltrating immune cells was then analyzed (**Supplementary Table 2**). The results revealed that immunosuppressive M2 macrophages were enriched in the high CHMP4C expression group (**Figure 6B**). We then explored the relationship between CHMP4C and 49 immune checkpoints and found that CHMP4C had a negative correlation with most immune checkpoints (**Figure 6C**, p<0.001). Above findings suggested to us that the high expression level of CHMP4C in prostate cancer may represent a worse prognosis after immunotherapy.

### 3.7 Targeted drug and immunotherapy response prediction for CHMP4C

**Figure 7:**
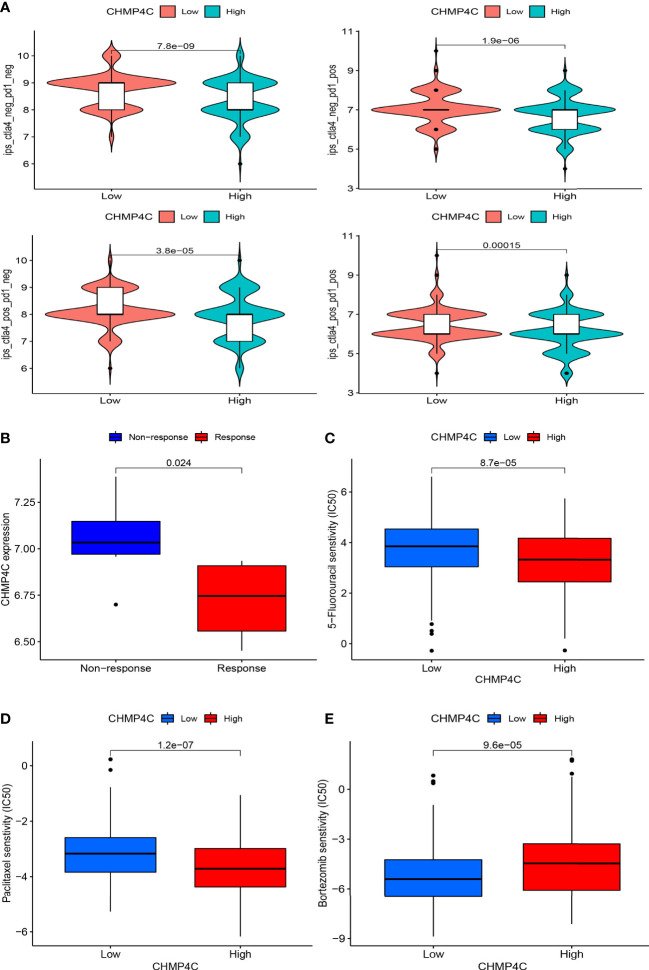
The immunotherapy and chemotherapy agents value of CHMP4C. Immune checkpoint inhibitor anti-PD1 and anti-CTLA-4 sensitivity analysis of four subtypes **(A)**. Prediction of sensitivity in an external PD-1/PD-L1 immunotherapy cohort **(B)**. Sensitivity analysis of 5-fluorouracil **(C)**, paclitaxel **(D)**, and bortezomib **(E)**.

The application of immune checkpoint inhibitors (ICI) has achieved success in tumor immunotherapy. To forecast the response to ICI, we computed scores for four subtypes based on machine learning. The results suggested that ​the group with lower expression of CHMP4C was more likely to respond to anti-PD1, anti-CTLA-4, and comprehensive therapy (**Figure 7A**). Combined with the above study, we concluded that the CHMP4C low expression group had better effect on immunotherapy and more clinical benefits for patients. The GSE67501 cohort is often used as an external independent cohort to assess immunotherapy effects (22). The predictive value of CHMP4C expression was further validated in an external cohort of 11 renal cell carcinoma patients who received PD-1/PD-L1 immunotherapy (GSE67501). The ‘stat_compare_means’ function showed that the patients with low CHMP4C expression exhibited a significantly higher response rate to the therapy (**Figure 7B**). We then utilized the R package ‘pRRophetic’ to predict vitro drug sensitivity according to the expression level of CHMP4C. The results suggested that the group with high expression of CHMP4C could benefit better when treated with paclitaxel and 5-fluorouracil (**Figures 7C, D**). Further analysis showed that bortezomib had lower IC50 values in the low expression group of CHMP4C, revealing that the bortezomib was more effective in patients with low CHMP4C (**Figure 7E**).

## 4 Discussion

Prostate cancer is the second most prevalent tumor in men, with a 5-year survival rate of 60% in Asia. In the United States, the 10-year survival rate for localized prostate cancer is nearly 100%. However, when distant metastasis occurs, the 5-year survival rate is only 32.3% (23) (24). Globally, the incidence of prostate cancer increased from 30.5 per 100,000 people in 1900 to 37.9 per 100,000 people in 2017 (25). Most prostate cancer will subsequently progress to the castration-resistant stage and result in the eventual death of prostate patients. Therefore, as a global health problem, prostate cancer seriously endangers the physical and mental health of men. Although PSA is the preferred serum marker for prostate cancer, it is not clear whether PSA is effective in reducing the risk of death in patients with prostate cancer (26). Hence, the search for biomarkers for the diagnosis, prognosis and treatment of prostate cancer is urgent. Pyroptosis as a form of programmed cell death plays an important role in the regulation of immune and inflammatory responses. Previous studies have demonstrated that pyroptosis-related genes can be used as new diagnostic and prognostic markers for tumors and contribute to the sensitivity analysis of immunotherapy, especially CHMP4C has an important role in cervical cancer, hepatocellular carcinoma and bladder cancer prognostic models (27) (28) (29). Therefore, the diagnostic, prognostic, and therapeutic value of CHMP4C in prostate cancer also deserves to be fully explored. The ESCRT mechanism (the endosomal cell sorting complex for translation) was involved in the normal separation of the genetic material of daughter cells during normal cell division. CHMP4C, as a component of endosomal sorting complex required for transport III (ESCRT-III), checked cell kinetics shedding by abscission checkpoint (11). The above mechanisms prevented excessive accumulation of DNA damage. In the absence of CHMP4C, cell shedding checks failed and damaged cells rapidly progressed from M to S phase, resulting in accumulation of DNA damage and genomic instability (12).

Our work explored the diagnostic, prognostic and therapeutic value of CHMP4C in the prostate cancer. We initially used the TCGA and TIMER databases to pinpoint the high expression of CHMP4C in prostate cancer, and we then confirmed the high expression of target genes at the molecular and protein levels. We then found that the CHMP4C expression level was significantly related to gleason score and lymph node status and at the same time high expression of CHMP4C was associated with poor prognosis. Subsequently, it was demonstrated that CHMP4C was highly expressed in the prostate cancer and was related to the advances in malignant biology. Moreover, *in vitro* prostate cancer cell growth, invasion, and metastasis were all considerably reduced when CHMP4C was knocked down. The above results fully illustrated that CHMP4C could be regarded as a novel diagnostic and prognostic marker in prostate cancer. CHMP4C has been shown to be up-regulated in a variety of tumors and is related to malignant behavior. CHMP4C as a model gene for pyroptosis was more closely associated with prostate cancer prognosis implying prognostic value of CHMP4C in prostate cancer (14). High enrichment of CHMP4C in the urine of patients with high Gleason score prostate cancer suggested the potential of CHMP4C as a novel diagnostic marker for prostate cancer (15). Among other cancers, CHMP4C was up-regulated in lung cancer and regulated tumor proliferation by modulating cell cycle progression (12). Meanwhile, high expression of CHMP4C also increased cell viability and anti-apoptosis in lung cancer under radiation conditions (11). Increased expression of CHMP4C in cervical cancer facilitated cervical cancer cell proliferation and invasion (13). Pancreatic cancer cell growth and invasion were markedly reduced when CHMP4C was knocked down (30). These results demonstrated the role of CHMP4C as an oncogene in tumors and were consistent with our findings.

To further explore the oncogenic role of CHMP4C, we performed a co-expression analysis using the ggplot2 and ggExtra R packages. We found that some of these genes are involved in cell cycle regulation especially CDK2 protein, suggesting that CHMP4C may affect prostate cancer progression in part by regulating the cell cycle. The results of GSEA analysis in GOBP and KEGG were also similar with the above findings. According to reports, CDK2 and cyclinA2 have significant regulatory functions in cell cycle and proliferation (31). This further validated the role of CHMP4C as a member of ESCRT in the regulation of cell cycle and proliferation and was consistent with our conjecture. Knockdown of CHMP4C led to reduced expression of CDK2 and cyclinA2. The above results demonstrated that CHMP4C partially mediated the regulation of cell proliferation by regulating the cell cycle.

Importantly, our subsequent GO and KEGG analysis of CHMP4C grouped differential genes revealed that CHMP4C may have been involved in the regulation of immune function in prostate cancer. Immunotherapy had bright promise in the treatment of prostate cancer (32). Regulation of immune function by CHMP4C may contribute to our understanding of immunotherapy in prostate cancer. We already know that the tumor microenvironment affected the efficacy and acted a crucial regulatory function in immunotherapy (33). We found that high expression of CHMP4C in tumor environment tended to have a lower immune score suggesting less lymphoid T-cell infiltration. This result suggested that high expression of CHMP4C had an immunosuppressive microenvironment compared to low expression groups. In the following immune infiltration cell analysis, we also found that macrophages M2 were abundantly enriched in the CHMP4C high expression group. Macrophage M2 promoted tumor cell development and metastasis and promoted tumor angiogenesis leading to tumor progression. Meanwhile, it also inhibited the T cell-mediated anti-tumor immune response (34). Above results suggested that CHMP4C may act as an immunosuppressive role in prostate cancer. In terms of immunotherapy, great progress has been made through immune checkpoint inhibitors (ICI) in urinary tumors, including kidney, bladder, and prostate cancers (35). However, the efficacy of ICI is not always satisfactory, and the degree of T-cell infiltration affects the final outcomes. Hot tumors with a large infiltration of T cells have a stronger response to ICI, while cold tumors are the opposite (36). In prostate cancer, immunosuppressive microenvironment and cold tumors were common immune features of prostate cancer and the efficacy of ICI was limited to specific subtypes of prostate cancer (35) (37). Therefore, immunotherapy for prostate cancer should be precisely classified to achieve maximum clinical benefit for prostate cancer patients. Our study proposed that CHMP4C based on low expression may have a stronger response to ICI to achieve precision therapy and improved the efficacy of ICI in prostate cancer. In our research, we discovered that CHMP4C was negatively related to most of the immune checkpoints. This result suggested that ICI might not be sensitive to the high expression group of CHMP4C. To confirm our above conjecture, we used an external database to predict the immunotherapeutic value of CHMP4C. CTLA4 and PD1 are currently the common immune targets in our immunotherapy, and the combination of both blockers has shown good efficacy in cancer immunotherapy (38) (39). In the clinical prediction of different treatment regimens for prostate cancer anti-PD1 and anti-CTLA4, the low CHMP4C expression group always showed better response compared to the high CHMP4C expression group. GSE67501 has been used as an independent cohort for immunotherapy (PD-1/PD-L1) to predict the value of target genes in immunotherapy (40) (41). In the GSE67501 cohort, the low expression CHMP4C group was also more sensitive to immunotherapy, which is consistent with our findings. In summary, the low-expression group of CHMP4C has a higher immune score and a more active immune microenvironment in prostate cancer, which is more favorable for immunotherapy of prostate cancer. In terms of other chemotherapeutic drug treatments *in vitro*, the treatment of paclitaxel and 5-fluorouracil has been very successful in prostate cancer (42). We predicted the sensitivity of paclitaxel and 5-fluorouracil according to the expression level of CHMP4C. Interestingly, the high CHMP4C expression group has a higher sensitivity to paclitaxel and 5-fluorouracil. Therefore, we can classify prostate cancer into different groups according to the expression of CHMP4C and adopted different treatment plans for the low and high expression groups of CHMP4C to achieve precise treatment of prostate cancer and maximize the clinical benefit of prostate cancer patients. However, our research still has certain shortcomings. Our analysis is mainly based on bioinformatics analysis and only a small amount of experimental verification has been carried out. Therefore, further research is required in the future to understand the precise mechanisms by which CHMP4C regulates the cell cycle and influences immunotherapy response in prostate cancer.

## 5 Conclusion

CHMP4C is highly expressed in prostate cancer tissues and plays a role in CHMP4C cell proliferation and metastasis by regulating the cell cycle. Importantly, CHMP4C is closely correlated with prostate cancer clinicopathological parameters and prognosis, indicating that CHMP4C can be used as a novel diagnostic and prognostic molecular marker for prostate cancer. Meanwhile, the expression can help to predict immunotherapy response in prostate cancer and implement different therapeutic regimens to achieve clinical benefit for prostate cancer patients.

## Data availability statement

The datasets presented in this study can be found in online repositories. The names of the repository/repositories and accession number(s) can be found in the article/**Supplementary Material**.

## Ethics statement

The studies involving human participants were reviewed and approved by The Medical Ethics Committee of the Second Hospital of Tianjin Medical University. The patients/participants provided their written informed consent to participate in this study.

## Author contributions

ZH researched and designed all bioinformatics analyses. DL, BY, LZ did the basic experimental part, and SX, DL, ZQ, SY and KW critically reviewed the manuscript. YX, HZ, KY and RL provided administrative, technical, and material support. All authors contributed to the article and approved the submitted version.

## Supplementary material

Supplementary Table 1 co-expression analysis of CHMP4C.

Supplementary Table 2 the correlation between CHMP4C and 22 infiltrating immune cells
